# Novel insights into exhaustive exercise-induced myocardial injury: Focusing on mitochondrial quality control

**DOI:** 10.3389/fcvm.2022.1015639

**Published:** 2022-10-14

**Authors:** Mingyue Shi, Zhao Dong, Kai Zhao, Xiaole He, Yang Sun, Jun Ren, Wei Ge

**Affiliations:** ^1^Department of General Practice, Xijing Hospital, Fourth Military Medical University, Xi'an, China; ^2^Department of Cardiology, Shanghai Institute of Cardiovascular Diseases, Zhongshan Hospital, Fudan University, Shanghai, China

**Keywords:** mitochondrial quality control (MQC), exhaustive exercise (EE), myocardial injury, mitochondrial dysfunction, mitophagy

## Abstract

Regular moderate-intensity exercise elicits benefit cardiovascular health outcomes. However, exhaustive exercise (EE) triggers arrhythmia, heart failure, and sudden cardiac death. Therefore, a better understanding of unfavorable heart sequelae of EE is important. Various mechanisms have been postulated for EE-induced cardiac injury, among which mitochondrial dysfunction is considered the cardinal machinery for pathogenesis of various diseases. Mitochondrial quality control (MQC) is critical for clearance of long-lived or damaged mitochondria, regulation of energy metabolism and cell apoptosis, maintenance of cardiac homeostasis and alleviation of EE-induced injury. In this review, we will focus on MQC mechanisms and propose mitochondrial pathophysiological targets for the management of EE-induced myocardial injury. A thorough understanding of how MQC system functions in the maintenance of mitochondrial homeostasis will provide a feasible rationale for developing potential therapeutic interventions for EE-induced injury.

## Introduction

Ample clinical and experimental evidence has shown a beneficial health outcome of regular moderate-intensity exercise on incidence of obesity, hypertension, cardiovascular disease (CVD), and certain cancers. The latest WHO guidelines on physical activity recommend at least 150–300 min of moderate-intensity aerobic activity or 75–150 mins of vigorous physical activity per week, sparking interest in exercise. Nonetheless, increasing the duration and intensity of exercise without restriction may be harmful to the human body. Exhaustive exercise (EE) refers to durable exercise loads beyond the bearing ability of the body (including overtraining in both competitive and recreational sports), which can cause physical fatigue and damage to multiple organs such as skeletal muscle, heart, liver, and kidneys. It is considered a pathological condition for multiple organs that imposes a wide variety of health hazards, such as heart abnormalities, chronic fatigue syndrome (CFS), and muscle degeneration (1–3). Although others have attempted to provide standardized definitions of EE intensity, a consensus has not been reached (4). As examples of EE, marathons, ultramarathons, and triathlons have gained increasing popularity worldwide, but physical injuries and even sudden deaths in these sports have raised some concerns. EE can cause damage to multiple organs, including skeletal muscle, heart, liver, and kidneys.

The heart is one of the organs most sensitive to EE. Young competitive athletes are reported to train for an average 10–20 h per week, in which case the cardiovascular system performs at a level 5–6 times greater than resting level, imposing a huge burden on the heart (5). It has been demonstrated that EE adversely impacts cardiovascular system, resulting in cardiac function decline, myocardial fibrosis and hypertrophy, arrhythmia, heart failure and even sudden cardiac death (SCD) (6, 7). For example, Chang and team proposed that EE may induce myocardial fibrosis, leading to ventricular hypertrophy, ischemic cardiomyopathy, arrhythmia and other undesired events for cardiovascular system (8). Athletes are reported to be four to eight times more likely to develop atrial fibrillation than the general population. Approximately, 12% of athletes develop atrial fibrillation (9). Baldesberger and coworkers found that professional cyclists possessed significantly higher rates of arrhythmias, particularly atrial fibrillation (AF) and atrial flutter (AFI) (10). Another survey showed the highest incidence of SCD among competitive athletes (ages 12–17), with 1.17 cases per 100,000 athletes annually (11). These data have cumulatively sparked major concerns regarding EE-induced myocardial injury.

Accumulating evidence has noted profound adverse cardiac effects of EE. EE can reduce excitability of cardiac autonomic cells and the velocity of sinoatrial conduction and atrioventricular conduction and prolong depolarization and repolarization of the atrium and ventricle, thus resulting in myocardial ischemia and arrhythmia. Long-term training and participation in extreme endurance competitions may lead to a transient reduction in cardiac ejection fraction and an increase in myocardial cell damage markers (12). After sustained exhaustive swimming, rats showed elevated levels of cardiac biomarkers cTnT (cardiac troponin T) and creatine kinase (CK) in the plasma, localized cardiomyocyte damage, functional impairment, and upregulated expression of malondialdehyde (MDA) in myocardia were observed. Simultaneously, the expression of matrix metalloproteinases was dysregulated, which was closely related to myocardial pathological damage and decreased cardiac function (13). In addition, EE also leads to myocardial ultrastructural destruction, abnormal energy metabolism, and mitochondrial dysfunction (14, 15). In animal experiments, EE rats exhibited characteristic myocardial changes, including myocardial nuclear matrix edema, enlargement of the nuclear space, decreased mitochondrial numbers, glycogen loss, and muscle fiber necrosis (16). Huang et al. observed a decrease in the left ventricular ejection fraction and an increase in the left ventricular systolic and diastolic volumes in rats after endurance training using echocardiography. Cardiac hypertrophy and increased cardiac weight occur simultaneously (17). These injuries are mainly due to abnormal mitochondrial metabolism, which in turn leads to an increased production of free radicals. Schoepe and team also found that myocardial mitochondria are an important site of EE-induced injury (18).

Multiple studies have depicted a significant role of mitochondria in EE-induced injuries. Thus, it is fundamental to study the mechanism of mitochondrial function during EE. In this study, we explore the relationship between mitochondrial quality control (MQC) and mitochondrial metabolism and their interaction with EE- induced myocardial injury will be explored. We then outline the possible therapeutic strategies and signal transduction pathways.

## Pathophysiology of mitochondria in EE-induced myocardial injury

The heart is composed of many cell types, including cardiomyocytes, fibroblasts, endothelial cells and pericytes, among which cardiomyocytes account for 75% of the total volume of the heart. The heart is a muscular organ working continuously for 24 h. Approximately 6 kg of adenosine triphosphate (ATP) is required to supply an essential energy basis for 24 h. Mitochondria are vital intracellular organelles that generate ATP to meet energy metabolic demands and maintain Ca^2+^-dependent contractions in cardiomyocytes. Cardiomyocytes demand high energy because of their contractility and rhythmic properties; therefore, they are enriched in mitochondria (35% of cell volume). During vigorous exercise, cardiomyocyte oxygen consumption increases by nearly 10 times (19). Mitochondrial dysfunction has been shown to compromise bioenergetics, metabolic signaling, apoptosis and cell death (20). Structural and functional integrity of mitochondria is essential for maintaining cardiac function (21).

Increasing evidence has depicted a rather important role of oxidative stress, proinflammatory response and apoptosis in EE-induced myocardial injury. Oxidative stress is caused by the overproduction of reactive oxygen species (ROS). Formation of ROS during exercise involves multiple mechanisms, including electron leakage from the mitochondrial respiratory chain, enhanced activities of NADPH oxidase and xanthine oxidase, increased free iron concentration, inhibition of mitochondrial uncoupling proteins (UCPs), and destruction of Ca^2+^ homeostasis. Among them, the mitochondrial respiratory chain is considered to be the cardinal source of exercise-induced ROS production, prompting an overt increase in ROS, accompanied by a loss of ATP synthesis (22). Superoxide dismutase (SOD) is an important antioxidant enzyme that repels ROS. MDA is the end-product of membrane lipid peroxidation, which directly causes membrane damage. EE increases oxygen consumption by cardiomyocytes, perturbing the redox balance in cells, and then leading to the aggregation of ROS in the body. Excessive ROS production initiates lipid peroxidation, protein oxidation, and cytoplasmic Ca^2+^ accumulation. Overtly decreased SOD levels lead to the generation of MDA, prompting cellular death and cardiac dysfunction. In addition, calcium overload activates Ca^2+^-dependent proteases, resulting in increased ROS production. Excessive cytoplasmic calcium deposition in mitochondria damages their structure and function (23). EE increases the release of inflammatory cytokines such as IL-6, IL-8, and IL-1β, and then activates the inflammatory response, which is another mechanism of EE-induced myocardial injury (24). NF-κB, a major regulator of inflammation, regulates the expression of various downstream genes. Activation of NF-κB leads to an increase in inflammatory proteins, such as TNF-α, IL-1, IL-6, and COX-2, and downregulation of the anti-inflammatory factor IL-10, resulting in cardiomyocyte proliferation, hypertrophy, and interstitial fibrosis (25, 26). Inflammation and oxidative stress are associated with heart disease. The occurrence of inflammatory response also leads to an increase in ROS levels in the body. Apoptosis is another major mechanism involved in EE and subsequent pathological changes. Oxidative stress triggers activation of apoptosis signaling, upregulates apoptosis-related regulators, and eventually induces myocardial injury (27, 28). Additionally, ROS can also negatively affect nuclear DNA (nuDNA) and mitochondrial DNA (mtDNA), leading to DNA strand breaks and point mutations. NuDNA encodes the replicase of mtDNA, and damage to the gene encoding this replicase affects mtDNA replication. Furthermore, mtDNA is close to the site of free radical generation, making it more likely to cause damage. These changes may reduce mitochondrial biogenesis, downregulate gene levels, alter Ca^2+^ and proton flux, and inevitably lead to organelle degeneration (29). Elevated intracellular Ca^2+^ levels, together with other factors, lead to increased oxidative stress and the opening of mitochondrial permeability transition pores (MPTP), a process that releases proapoptotic compounds and subsequently activates caspases, resulting in mitochondrial swelling, tissue damage, and myocardial apoptosis (30).

As mitochondria are finely regulated by a variety of regulatory molecules and exert a strong influence on life activities, there is an urgent need to elucidate their quality control mechanisms in detail to protect the myocardium from dysfunctional mitochondria.

## MQC in EE-induced myocardial injury

Under physiological conditions, mitochondria maintain dynamic changes in morphology and structure through biogenesis, fusion and fission, mitophagy and mitochondria-mediated cell death; this process is known as MQC (31). Moderate exercise maintains mitochondrial function through the coordinated regulation of mitochondrial biogenesis and mitochondrial fusion, and fission. Appropriate MQC activates apoptosis, inflammatory pathways, and selective autophagy, thereby facilitating the clearance of damaged mitochondria (32). Nevertheless, EE leads to disturbance of the MQC axis, resulting in decreased mitochondrial respiratory function, excessive activation of apoptotic pathways, damage to the myocardial structure, and cardiac dysfunction. However, the detailed mechanism of MQC in EE-induced myocardial injury remains unclear. Therefore, an in-depth understanding of the mitochondrial response to EE and the regulation of MQC will help determine the treatment of EE-induced myocardial injury.

### Role of mitochondrial biogenesis in EE-induced myocardial injury

Mitochondrial biogenesis is influenced by multiple factors, such as oxidative stress, exercise training, myocardial infarction, cardiac fibrosis, and other environmental stresses (33). Mitochondrial biogenesis alters mitochondrial content and regulates synthesis of new mitochondria, which requires the participation of both mitochondrial and nuclear genomes. This system is regulated by several processes, including the generation of mitochondrial outer and inner membranes, replication of mtDNA, and synthesis of protein input in the cytoplasm and transport to proper inner mitochondrial regional compartments (outer mitochondrial membrane, intermembrane space, inner mitochondrial membrane, and mitochondrial matrix) (34). The peroxisome proliferator-activated receptor (PPAR) gamma transcriptional co-activator PGC-1 family (PGC-1α and PGC-1β) are the most important initiating factors. PGC-1α upregulates the expression of nuclear respiratory factors (NRF1 and NRF2) and binds to them, activating multiple nuclear encoding genes and Tfam, which regulates mitochondrial transcription. NRF1 and NRF2 participate in the synthesis of mitochondrial respiratory complex subunits and regulate cellular respiration. Tfam controls mtDNA replication and transcription and maintains its integrity (35). Overexpression of PGC-1α is correlated with increased levels of nuclear-encoded mitochondrial genes, which enhances the ability of mitochondria to undergo oxidative phosphorylation and leads to mitochondrial biogenesis, driving the synthesis of mitochondrial DNA, proteins, and generation of new mitochondria.

**Figure 1:**
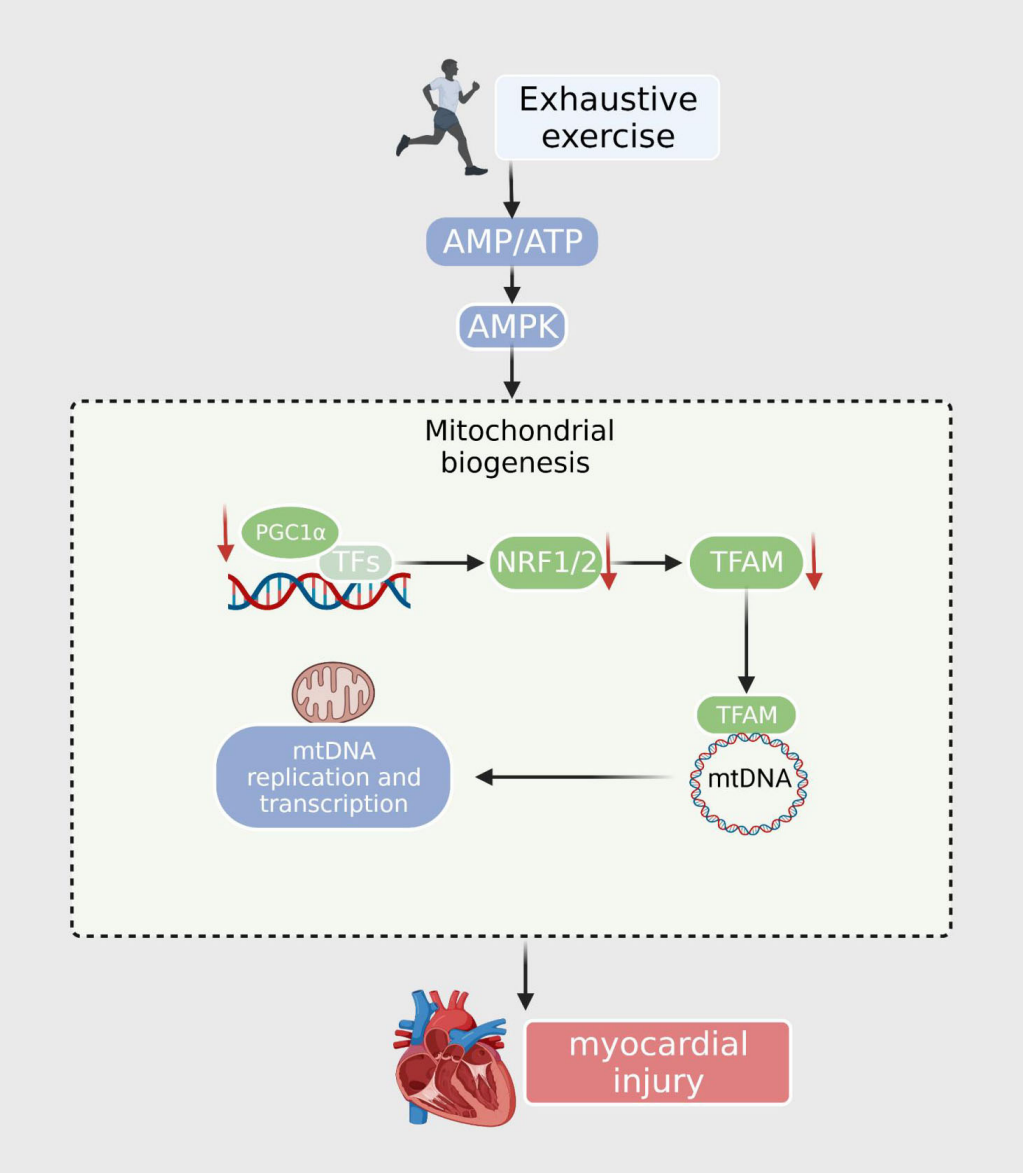
After exhaustive exercise, the expression of PGC-1α and other transcription factors (NRF1, NRF2) is downregulated. The TFAM is then reduced when imported into the mitochondria. TFAM downregulates the expression of nuclear encoding genes, resulting in decreased mtDNA synthesis, ATP synthesis, and mitochondrial content.

PGC-1α is essential for exercise-induced mitochondrial biogenesis. Knockdown of PGC-1α may cause stalled mitochondrial turnover and death in cardiomyocytes, thereby affecting cardiac function and leading to a reduction in exercise-induced metabolic benefits (36). EE leads to a marked downregulation of PGC-1α and mitochondrial respiratory chain complexes I and II in the heart, along with a reduction in mtDNA copy number (37). A drop in mitochondrial complex I and II activity would suppress mitochondrial oxidative phosphorylation and increase ROS yield. Mille-Hamard observed a decrease in PGC-1α mRNA expression after EE, and the exact mechanism remains unclear (38). The PGC-1α-NRF1/Nrf2-TFAM signaling pathway, the key mitochondrial biogenesis regulatory factor, is responsible for energy production. Studies have found that PGC-1α and NRF levels in the EE murine model were significantly reduced, and the hypoxic environment caused by EE also led to a decrease in Tfam expression. These results demonstrated that EE interferes with the synthesis of the mitochondrial respiration complex and decreases the activity of gene regulation by inhibiting the PGC-1α-NRF1/Nrf2-TFAM signaling pathway (16); This may lead to mitochondrial energy supply disorders and insufficient myocardial oxidative capacity and energy production, resulting in irreversible myocardial damage (39). However, the detailed mechanism by which mitochondrial biogenesis dysfunction contributes to EE requires further exploration (Figure 1).

### Involvement of mitochondrial dynamics in EE-induced myocardial injury

Ample evidence has suggested that mitochondria undergo continuous fusion and fission to maintain mitochondrial integrity and remove damaged mitochondria (40). The dynamic balance between fission and fusion contributes to the maintenance of the morphology and function of intracellular mitochondrial networks, as well as the dynamic changes in mitochondria and energy metabolism under various stress conditions, promoting cell development and cell death (41). Giant mitochondria were found in an ultrastructural analysis of left ventricles of physically trained mice, indicating the presence of multiple fusion events.

Mitochondrial fusion promotes the formation of elongated or reticular mitochondria essential for regulating cardiomyocyte homeostasis (42). Mitofusins (Mfn1 and Mfn2) and optic atrophy protein 1 (OPA1) are the radical effectors of mitochondrial fusion in mammalian cells. Mfn1/2 can regulate the fusion of the outer mitochondrial membranes, whereas Opa1 is involved in the regulation of the inner mitochondrial membrane and cristae (43). Low expression of Mfn1/2 can lead to a decrease in mitochondrial fusion efficiency and fragmentation and interference with the mitochondrial energy supply to cells. Inhibition of Mfn1/2 can promote apoptosis, whereas overexpression of Mfn1/2 promotes mitochondrial fusion and alleviates mitochondrial dysfunction. Mitochondrial fission is mediated by coordinated and synergistic interplay among various mitochondrial fission proteins (Drp1, Fis1, Mff, and Mid49/51) (44). Translocation of Drp1 from cytoplasm to the outer mitochondrial membrane induces mitochondrial fission. DRP1 is recruited from the cytoplasm to the mitochondria. In addition, the formation of circular polymers with MID49/51, Fis1, and Mff results in GTP hydrolysis and mitochondrial microtubule redistribution, a process that produces fragmented discrete organelles (45). Furthermore, some more proteins may also be indispensable for the regulation of mitochondrial fission (46). When the damaged mitochondria are generated by mitochondrial fission, the activity of mitophagy is enhanced, so that the damaged mitochondria are degraded in time (47, 48). However, excessive fission leads to decreased ATP synthesis, excessive oxidative stress, and apoptosis.

**Figure 2:**
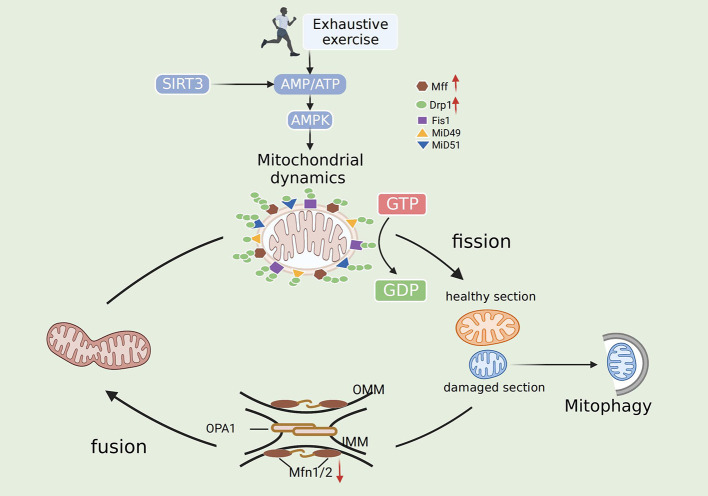
Mechanisms of mitochondrial dynamics after exhaustive exercise. In general, fission is mediated by Drp1, Drp1 receptors (Mid 49, Mid51, Mff) and Fis1. Fusion is mediated by Mfn1, Mfn2 and Opa1. Fission and fusion machineries regulate mitochondrial by dislodging the damaged organelles. Exhaustive exercise disbalances mitochondrial fission and fusion, and the mitochondrial network is gradually impaired. The mitochondria undergo both symmetrical and asymmetrical fission. Symmetrical fission generates two “new” healthy mitochondria, whereas asymmetrical fission of mitochondria produces healthy and damaged sections. The healthy section fuses with other healthy organelles for regeneration. The damaged section can be cleared by autophagosomal engulfment, thereby protecting the cell from mitochondrial toxicity.

Emerging data suggest that fission/fusion proteins are critical for myocardial energy metabolism, and their dysfunction contributes to various cardiac diseases. EE involves mechanisms related to acute ischemic stress (49). Oxidative stress-mediated downregulation of Mfn1/Mfn2 leads to mitochondrial morphological disorders and fragmentation (50). Zhou and colleagues indicated that content of the mitochondrial fission protein Mff is significantly increased after I/R, leading to the impairment of mitochondrial structure and function by increasing mitochondrial fission and diminishing mitochondrial fusion (51). Samant and associates indicated that the nuclear NAD-dependent histone deacetylase Sirtuin-3 (Sirt3) binds to OPA1 to foster its activation. Mitochondrial fusion is reduced in Sirt3-knockout adult cardiac fibroblasts (52). Moreover, overexpression of Sirt3 inhibits mitochondrial fission through the AMPK-Drp1 pathway in cardiovascular disease, thus promoting ROS scavenging and reducing inflammation and cellular damage (53). EE decreases the number of myocardial mitochondria and the mass of the myocardium by downregulating Mfn2 and increasing Drp1, resulting in energy metabolism imbalance and myocardial damage. In addition, the expression of Mfn1 and OPA1 was not affected by EE (37, 54). Another study illustrated that phosphorylation of Drp1, but not Drp1, affects mitochondrial fission during EE. During EE, mitochondrial fission leads to a large number of mPTP openings, and certain substances enter mitochondria, causing mitochondrial edema. Meanwhile, pro-apoptotic substances are released into the cytoplasm, causing apoptosis and mitochondrial dysfunction (55). Summarizing the experiments of many researchers, Atkins concluded that Mid49/51 is expressed at the highest levels in the heart, promoting fusion and fission. This finding further supports the importance of MiD proteins in the mechanism of myocardial mitochondrial dynamics (56). Therefore, mitochondrial dynamics may serve as an important target for the treatment of EE-induced myocardial injury (Figure 2).

### Mitophagy pathways and role in EE-induced myocardial injury

**Figure 3:**
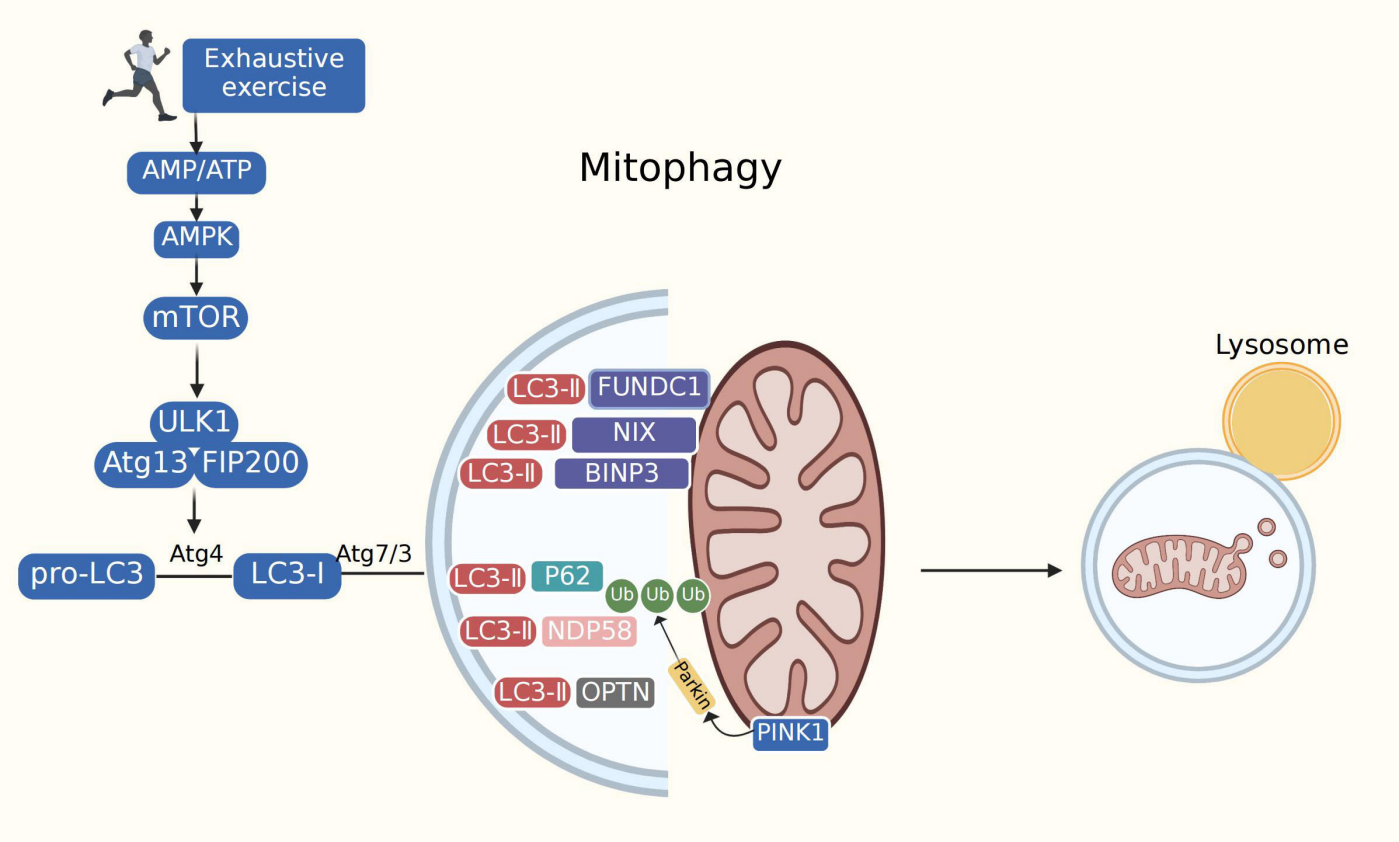
Mitophagy. Mitophagy is mediated by many of cellular signal mechanisms including PINK1, Parkin, mitophagy receptors (BNIP3L/NIX, FUNDC1), as well as certain mitophagy adaptors. Adapter proteins (p62, OPTN, NDP58) recognize phosphorylated polyubiquitin chains on mitochondrial proteins and initiate autophagosome formation by binding to LC3. The exact role of mitophagy in exhaustive exercise remains controversial. Most studies have depicted suppressed autophagy during exhaustive exercise. However, several research have found excessive autophagy in exhaustive exercise. Mitophagy impairment may lead to the accumulation of dysfunctional mitochondria, prompting oxidative stress, mitochondrial dysfunction and cardiac injury.

Mitophagy is the selective removal of damaged or malfunctioning mitochondria by cells using lysosomes to reduce unnecessary cell death. In the physiological environment, mitophagy regulates the number of intracellular mitochondria and maintains their normal function. Our team showed that mitophagy is involved in the pathogenesis of various cardiovascular and metabolic diseases, including aging, obesity, insulin resistance, diabetes mellitus, atherosclerosis, and ischemic stroke (40, 57–60). Our results demonstrated that mitophagy is the most important component of the MQC system and the primary safeguard for mitochondrial homeostasis and integrity. Excessive mitophagy results in mitochondrial dysfunction and cell demise (31). Many crucial genes (autophagy-related genes, ATGs) and proteins are involved in autophagy initiation and regulation. The three most studied pathways are PINK1/Parkin, BNIP3, and FUNDC1. Research from our laboratory has revealed that FUNDC1 is indispensable in pathological diseases, such as heart failure and ischemia-reperfusion injury (61). P62 is an essential chaperone that mediates parkin-dependent mitophagy. P62 levels are inversely correlated with autophagic activity (62). Other proteins, including UNC-51 autophagy-activating kinase 1 (Ulk1) and LC3, also have biological functions in regulating autophagy. Ulk1, present in a complex containing FIP200, Atg13, and ATG101, initiates autophagosome formation by phosphorylating beclin-1. MTORC1 inactivates these autophagic regulatory complexes, thus affecting autophagy (63, 64). LC3 is cleaved into LC3-I by the autophagy-specific gene *Atg4*, and LC3-I is modified and processed by ubiquitin-like bodies, including Atg7 and Atg3, to generate LC3-II, which is localized to autophagosomes. This process turns off the autophagosomes and transports intact autophagosomes to the lysosomes for degradation. Both BNIP3L/NIX and FUNDC1 interact with LC3 to recruit autophagosomes and mediate mitophagy (65). The heart was confirmed to be the most robust mitochondrial phagocytic organ in mito-Keima-expressing mice, indicating the essential role of mitophagy in the occurrence and development of cardiovascular disease (66) (Figure 3).

Ample evidence has implicated the role of mitophagy in the pathogenesis of EE-induced myocardial injury. For example, inhibition of LC3-II turnover results in the accumulation of LC3-II levels, LC3-II/LC3-I ratio, and p62 levels during EE, suggesting a block in autophagosome degradation during EE. The inhibition of autophagosome clearance can induce the accumulation of misfolded proteins and damaged organelles, exacerbating myocardial injury (67). Data obtained from a recent study showed alterations in autophagy levels by examining autophagy protein expression after EE. Researchers found that the LC3-II/LC3-I ratio was reduced during EE, but Beclin1 and p62 levels showed no obvious changes (13). Another study developed a model of EE-induced myocardial injury in which LC3-I could not be completely converted into LC3-II. The LC3-II and LC3-II/LC3-I ratios were found to be barely elevated, although the levels of p62 were elevated during EE (68). These data suggest that after EE, autophagy levels are inhibited, and the degradation of metabolically depleting proteins or damaged organelles is blocked, thus aggravating myocardial ischemia-hypoxia injury. However, there have been a few different results. Previous work confirmed that accumulation of autophagosomes and LC3-II turnover in EE group was overtly upregulated compared with other exercise groups. Excessive autophagy activated by EE may contribute to the onset of cardiomyocyte damage (69). Furthermore, AMPK-mTOR plays a pivotal role in autophagic initiation in cardiomyocytes (70). One study demonstrated that EE can cause myocardial injury through activation of the AMPK-mTOR-ULk1 signaling pathway. Overexpression of the AMPK-mTOR-ULk1 signaling pathway results in excessive mitochondrial autophagy and the autophagy-selective cell death “autosis” (71). Thus, the mechanism of autophagy during EE should be further investigated. Future studies should focus on the role of autophagy in EE-induced myocardial injury to ascertain possible targets for intervention.

### Mitochondria-mediated cell death in EE-induced myocardial injury

Apoptosis is an active cell death process regulated by a variety of genes that occurs under certain physiological or pathological conditions. Mitochondria play an irreplaceable and important role in apoptosis as a central link in the apoptotic pathway (72). Cytochrome C (Cyt C) is an apoptosis-regulated protein normally located outside the inner mitochondrial membrane. The stimulation of apoptotic signals causes Cyt C to be released into the cellular matrix, where it combines with other substances to form apoptotic bodies and trigger apoptosis. Cleaved caspase-3 and PARP are important markers of myocardial apoptosis. The release of Cyt C triggers a cascade of caspases, thereby inducing apoptosis. The B-cell lymphoma-2 (Bcl-2) family includes both anti-apoptotic Bcl-2 and pro-apoptotic Bax proteins associated with apoptosis. Bcl-2 family members are mainly located in the mitochondria and regulate the permeability and integrity of the outer mitochondrial membrane; however, the family members promoting apoptosis are mainly located in the cytoplasm. The Bax/Bcl-2 ratio determines the permeability of various mitochondrial membrane channels, thus affecting cell survival and death. After the cell receives the apoptosis signal, the pro-apoptotic factors Bax and Bak are ectopic in the mitochondrial outer membrane, and various proteins in the mitochondria are released, including the second mitochondrial-derived caspase activator (Smac), nuclease G, apoptosis inducible factor (AIF), and HtrA2. These are relocated to the nucleus, resulting in large-scale DNA fragmentation, chromatin condensation, and the activation of mitochondrial caspase-independent apoptotic pathways (73).

EE induced cardiomyocyte loss and increased left ventricular apoptosis, as manifested by increased levels of the Bax/Bcl-2 ratio, Cyt C, cleaved caspases-3, and PARP in the LV tissue of the rat model (74). EE resulted in a significant increase in the Bax/Bcl-2 ratio and the number of TUNEL-positive cardiomyocyte nuclei, enhancing apoptotic signaling in the myocardium, and thereby resulting in DNA fragmentation (10). Several studies have confirmed that EE significantly reduces the Bcl-2/Bax ratio and increases the levels of caspase-3 and caspase-9 and the number of apoptotic myocardial cells. These studies indicate that cardiomyocyte apoptosis is significantly increased by the action of mitochondria, which is an important mechanism of EE-induced myocardial injury (26, 75, 76). Oxidative stress and inflammatory responses can promote apoptosis. EE promotes the production of excessive ROS in the myocardial mitochondria. ROS induce the recruitment of apoptosis-related speck-like proteins and activation of caspase-1. This process disposes of IL-1β precursors and secretes mature IL-1β, which further amplifies the inflammatory response, ultimately leading to abnormal mitochondrial structure and programmed cell death (77). Apoptosis is a complex molecular regulatory mechanism. A recent study has revealed that the Nrf2/HO-1 signaling cascade inhibits oxidative stress and apoptosis. Increased levels of Nrf2, an important transcription factor regulating cellular redox homeostasis, act on HO-1 and further interact with other transcription factors to inhibit downstream proteins including Bax. EE downregulates Nrf2 and HO-1 proteins, upregulates mitochondrial apoptosis-related proteins, and induces apoptosis (26). Moreover, regulation of apoptosis *via* the PI3K-Akt pathway is complicated. Studies have confirmed that the activation of this pathway is related to cardiovascular diseases. Akt is an upstream signaling protein in the BCL-2 family. The PI3K-Akt signaling pathway can inhibit the opening of MPTP and the release of apoptotic factors from mitochondria to regulate cardiomyocyte apoptosis (78). Previous studies have found that myocardial fibers in EE mice exhibit tissue disorders, rupture, degeneration, and necrosis. The possible mechanism is that the activity of the PI3K/Akt pathway is inhibited, the level of anti-apoptotic proteins is downregulated, and the level of pro-apoptotic proteins is upregulated, thereby reducing the opening level of MPTP (1). These findings suggest that EE can weaken the defense capacity of myocardial cells and accelerate apoptosis, resulting in myocardial injury.

## Targeting MQC for management of EE-induced myocardial injury

### Prevention of mitochondrial injury by exercise preconditioning

Considerable research attention directed toward the possible applicability of exercise preconditioning (EP) for the treatment of EE-induced myocardial injury. EP refers to exercise intervention with appropriate intensity and repetitions, which can enhance myocardial tolerance to ischemia-hypoxia and induce myocardial protection (79, 80). EP can alleviate the degree of EE damage to the systolic and diastolic functions of rats, enhance myocardial antioxidant capacity, and improve myocardial function (1).

EP may prevent mitochondrial damage after EE through several potential mediators, such as heat shock proteins (HSP), Ca2^+^-handling proteins, and endogenous antioxidants (81). However, there are other mechanisms through which EP induces cardioprotection. Recently, investigators have shown that EP can upregulate the level of PGC-1α-Nrf1/Nrf2-TFAM, a key pathway of mitochondrial biogenesis, improve mitochondrial respiratory function, and enhance energy metabolism in cardiomyocytes. EP upregulated the expression of MFN2 and downregulated the expression of DRP1, reducing the fragmentation of myocardial mitochondria to a certain extent (82). Li found that several autophagy formation proteins (LC3-II/LC3-I ratio, Atg7, and Atg5) were elevated after EP, demonstrating that EP can maintain mitochondrial homeostasis and cell survival by promoting autophagosome formation and clearance, thereby preventing EE-induced myocardial injury (13). Some studies have confirmed that EP inhibits the decreased expression of Bcl-2 caused by EE through the activation of the PI3K-Akt pathway. Moreover, it contributed to the downregulation of the expression of Bad, Bax, and caspase-3 (1). In general, EP can induce myocardial protection after EE, reduce myocardial apoptosis, and improve the morphological structure of cardiomyocytes. Further research is needed to investigate the molecular mechanisms underlying EP.

### Prevention of mitochondrial injury following EE using pharmaceutical supplements

Individuals who exercise aggressively may benefit from supplementation with mitochondrial nutrients before exercise to minimize mitochondrial damage from EE. Moreover, mitochondrial nutrients or drugs can promote mitochondrial damage repair in individuals with mitochondrial damage following excessive exercise (83). Although there are currently no FDA-approved drugs for the treatment of EE-induced myocardial injury, several mitochondrion-targeted drugs are being explored, including CoQ10 and certain natural Chinese medicine ingredients with antioxidant effects, such as resveratrol, tetramethylpyrazine, and trimetazidine. These regulatory agents exhibit their underlying mitochondrial targeting mechanisms to modulate mitochondrial biogenesis, dynamics, mitophagy, and mitochondria-mediated cell death.

Okudan observed a decrease in EE-induced markers of oxidative stress in rat hearts following CoQ10 supplementation, suggesting that CoQ10 may increase antioxidant enzyme activity and reduce inflammatory markers, thereby protecting myocardial mitochondria from lipid peroxidative damage (84). Studies have found that resveratrol can induce mitochondrial biogenesis, inhibit excessive mitochondrial fission, improve fusion, and play a part in maintaining cardiac function and improving exercise capacity (85, 86). Furthermore, resveratrol induces mitophagy through the inhibition of mTORC1 (87). Research conducted by liu also indicated that 3-MA significantly decreased EE-induced elevation in LC3-II/I ratio and beclin-1, and alleviated EE-induced myocardial injury (69). Tetramethylpyrazine and trimetazidine preconditioning can facilitate Bcl-2 expression and decrease the expression of caspase-3 and caspase-9 to suppress excessive apoptosis in myocardial cells (76, 88). Regulating these molecules and reducing oxidative stress with mitochondria-targeting nutrients may be an effective strategy to treat EE-induced myocardial injury.

## Future perspectives

EE commonly occurs in professional athletes undergoing regular high-intensity training and is often overlooked. Accumulating evidence has shown that EE-induced myocardial injury may be an important risk factor for cardiovascular diseases, with multifaceted mechanisms of action, particularly mitochondrial dysfunction. MQC is a unique process that maintains mitochondrial integrity and homeostasis. However, the regulation of MQC, specific molecular targets involved, and their interaction are not understood. Therefore, further studies targeting mitochondria and examining MQC in exhausted animal models are needed to reveal new options to manipulate mitochondrial function and quality control and ameliorate oxidative and cellular damage caused by EE.

Several mitochondrion-targeted drugs, including those mentioned above, are under scrutiny to better understand the pathological basis for EE-induced myocardial injury. These drugs may be effective strategies for improving physical performance and injury recovery after EE. This is an important issue that should be addressed in future research.

In addition, different intensities of EP have different protective effects against EE-induced myocardial injury. It is also of great significance to explore the optimal intensity of EP training plans, formulate scientific and personalized exercise prescriptions, reasonably improve exercise intensity, and fully monitor exercise plans to prevent myocardial injury. Meanwhile, it is important to explore the pathogenesis, prevention, and treatment of EE-induced myocardial injury in combination with exercise type.

## Data availability statement

The original contributions presented in the study are included in the article/supplementary material, further inquiries can be directed to the corresponding author/s.

## Author contributions

MS and ZD drafted the manuscript. WG, JR, KZ, XH, and YS edited the manuscript. WG and JR put forward some amendments to the article. WG supervised the whole process of this review. All authors contributed to the article and approved the submitted version.

## Funding

This study was supported in part by the Natural Science Foundation of China (NSFC 82270259 and 81471409), Grants of the Major Science and Technology Project of Shaanxi Province (2017ZDXMSF023), and Promotion Program (XJZT19Z18, XJZT19X10, and KJYXJY2021021).

## Conflict of interest

The authors declare that the research was conducted in the absence of any commercial or financial relationships that could be construed as a potential conflict of interest.

## Publisher's note

All claims expressed in this article are solely those of the authors and do not necessarily represent those of their affiliated organizations, or those of the publisher, the editors and the reviewers. Any product that may be evaluated in this article, or claim that may be made by its manufacturer, is not guaranteed or endorsed by the publisher.

## Glossary

ROS, Reactive oxygen species; MQC, mitochondrial quality control; mtDNA, Mitochondrial DNA; cTnT, cardiac troponin T; SOD, Superoxide dismutase; nuDNA, nuclear DNA; mPTP, Mitochondrial permeability transition pore; IL-1α, Interleukin-1 alpha; MMP, mitochondrial membrane potential; NF-kB, nuclear factor kappa-B; PPAR, peroxisome proliferator-activated receptor; Drp1, Dynamin-related protein 1; PGC-1α, Peroxisome proliferator-activated receptor *γ* co-activator 1α; Tfam, Transcription Factor A Mitochondrial; Mff, Mitochondrial fssion factor; Mfn1, Mitofusin 1; Mfn2, Mitofusin 2; Fis1, Fission protein-1; MiD49, Mitochondrial dynamics proteins 49; ER, Endoplasmic reticulum; MiD51, Mitochondrial dynamics proteins 51; Opa1, Optic atrophy protein-1; IMM, Inner mitochondrial membrane; OMM, outer mitochondrial membrane; I/R, Ischemia/reperfusion; PINK1, Phosphatase and tensin homolog-induced kinase 1; Sirt3, Sirtuin 3; BNIP3, BCL2 and adenovirus E1B 19-kDa-interacting protein 3; FUNDC1, FUN14 domain containing 1; MAPK, Mitogen-activated protein kinase; VDAC1, Voltage-dependent anion-selective channel 1; LC3, Light chain-3 protein; mTORC1, Mechanistic target of rapamycin complex 1; AMPK, 5′-AMP-activated kinase; ULK1/2, Unc-51-like autophagy-activating kinase 1/2; PI3K/Akt pathway, Phosphatidylinositol-4,5-bisphosphate 3-kinase (PI3K)/protein kinase B (Akt) pathway; Cyt c, cytochrome c; Bcl-2, B-cell lymphoma-2; (SQSTM1, p62), Sequestosome 1; 3-MA, 3-Methyladenine; CoQ10, coenzyme Q10; FIP200, FAK family kinase-interacting protein of 200 kDa; HtrA2, hish temperature requirement A2; EP, Exercise preconditioning; Smac, second mitochondrial derived caspase activator.
